# π-Bridge Effect on Symmetric Carbazole-Based Small Molecules for Realizing Ultraviolet Fluorescent Emission

**DOI:** 10.3390/ma11040617

**Published:** 2018-04-17

**Authors:** Siyang Liu, Pengju Lin, Fangfang Niu, Pengju Zeng, Bin Zhang

**Affiliations:** 1 Key Laboratory of Optoelectronic Devices and Systems of Ministry of Education and Guangdong Province, College of Optoelectronic Engineering, Shenzhen University, Shenzhen 518060, China; ray-lsy@hotmail.com (S.L.); 2170285401@email.szu.edu.cn (P.L.); ffn@szu.edu.cn (F.N.); 2 School of Materials Science and Engineering, Jiangsu Engineering Laboratory of Light-Electricity-Heat Energy-Converting Materials and Applications, Jiangsu Key Laboratories of Environment-Friendly Polymers, National Experimental Demonstration Center for Materials Science and Engineering, Changzhou University, Changzhou 213164, China

**Keywords:** carbazole, symmetrical, π-bridge effect, ultraviolet emission

## Abstract

A series of symmetric carbazole derivatives (CzP-H, CzP-CN, CzP-Me, and CzP-OMe), which comprise electron-donating and electron-drawing groups appending on a phenyl core, was synthesized and characterized in detail. These compounds exhibit excellent thermal stabilities, with thermal decomposition temperatures exceeding 400 °C. From the fluorescent spectra in film, CzP-H, CzP-Me, and CzP-OMe showed UV to blue-violet emission, with peaks at 396 nm, 402 nm, and 392 nm, respectively. The *E*_00_ energies of CzP-H, CzP-CN, CzP-Me, and CzP-OMe were 3.39 eV, 2.83 eV, 3.50 eV, and 3.35 eV, respectively. From the electrochemical measurements, the highest occupied molecular orbital (HOMOs) energy levels were −5.30 eV, −5.64 eV, −5.46 eV, and −5.24 eV for CzP-H, CzP-CN, CzP-Me, and CzP-OMe, respectively. Through calculations from HOMO energy levels and *E*_00_ energies, the lowest unoccupied molecular orbital (LUMOs) energy levels of CzP-H, CzP-CN, CzP-Me, and CzP-OMe were −1.91 eV, −2.81 eV, −1.96 eV, and −1.89 eV, respectively. Therefore, the introduction of different substitutes in phenyl cores would distinctly affect the photophysical properties. These results indicate that the prepared carbazole derivatives could be potential candidates for realizing ultraviolet or blue-violet emission.

## 1. Introduction

Recently, organic electronics (OEs) are becoming more attractive due to their unique merits in low cost, lightweight, large-scale fabrication and flexibility. Until now, plenty of functional small molecules and polymers have been developed for optoelectronic devices, including organic light-emitting diodes (OLEDs) [1], organic solar cells (OSCs) [2], organic field-effect transistors (OFETs) [3] and organic photodetectors (OPDs) [4]. Among them, some are utilized dramatically in the market, ascribing to their versatile characteristics, such as OLEDs.

The technology of OLEDs is a promising solution for realizing full-color and high-resolution flat-panel displays and flexible devices. Since their discovery in 1987 [5], the stable and efficient ultraviolet emitters have become quite crucial for commercial applications. In general, three primary light emitters (red, green, and blue) have basically satisfied the commercial requirements for their electroluminescent devices with high efficiencies, long lifetimes, and pure Commission International de l’Eclairage (CIE) coordinates [6,7]. Although important progress has been achieved, there is increasingly urgency to design and manufacture much more extensive applications of OLEDs in medical and health facilities. Unlike present gas-based ultraviolet light sources, ultraviolet OLED emitters show versatile advantages such as their high efficiency, low-driving voltage, self-emitting properties, pure color, high brightness and environmentally-green characteristics [8,9]. Due to the tough requirements of the intrinsic wide band gaps in ultraviolet materials, weak conjugation [10] is commonly proposed for designing and preparing molecules; however, this could lead to low carrier mobility and difficult carrier injection from both anode and cathode electrodes [11,12]. As a result, the development of stable and high-efficiency organic ultraviolet materials in OLEDs is a significant issue.

Both the obvious shortages of traditional ultraviolet light apparatuses and the promising future of OLEDs in medical and health care have prompted research studies focused on developing efficient wide band-gap organic materials for realizing ultraviolet emission. In addition, the continual improvements of OLEDs provide steady backup for designing and preparing new ultraviolet materials. Even though organics-based ultraviolet materials possess these advantages, obtaining high-performance ultraviolet organic materials still faces lots of obstacles. The traditional deep-blue fluorophores, such as anthracene [13], aromatic hydrocarbons [14], dihydrodinaphthoheptacene [15], carbazole [16], fluorene [17], triphenylamine [18], phenylene [19], pyrene [20] and phosphorescent iridium complexes [21] showed good performance in dilute solution, but urgent demands for further improvements remain regarding ultraviolet color purity, intermolecular stacking, solid-state morphology, efficiency and device lifetime. For instance, the negative aggregation of molecules in the solid state could induce red-shifted emissions and aggregation quench, the consequences limit the realization of pure ultraviolet emission. Furthermore, some of these materials failed to provide dense films and the thin films tended to crystalize during deposition and produced the rough surface, grain boundaries or pin holes leading to current leakage. To achieve high-performance ultraviolet molecules, a number of small molecules and polymers have been designed and developed, including π-conjugated organics via modification of chemical structures, asymmetric non-planar molecules [22], donor-acceptor typed dopants [23], phosphorescent emitters [24], star-shaped polyfluorenes [25], cationic iridium complexes [26], dihydroindenofluorene [27] and monodisperse macromolecules [28]. However, most of them were not good enough for shorter wavelength emission with high color purity. Recently, a series of versatile star-shaped carbazole derivatives were reported, which comprised various aryl groups appending in the 1, 3, 6, and 8 positions of 9-alkyl-carbazole. These molecules exhibited the ultraviolet emission at about 394 nm with a narrow full width at half maxima (FWHM) of 42 nm, and versatile thermal stability with thermal decomposition temperatures ranging from 321 to 485 °C. However, these carbazole derivatives were still facing photoluminescence quantum yields (PLQY, *Φ*_f_) as low as 3.4% [29].

However, the new series of carbazole-based ultraviolet emitters in terms of larger band gaps, high quantum efficiency and high color purity remain to be explored [30,31,32,33]. Carbazole-based small molecules exhibit excellent performance in ultraviolet/deep-blue OLEDs; for example, they demonstrate large conjugation stability, high hole mobility and high thermal properties. However, they also suffer from some defects that lead to the limited application in OLEDs. Therefore, intense investigations have been carried out to improve the performance of carbazole-based ultraviolet molecules [34,35,36,37]. Herein, we designed a series of symmetric ultraviolet small molecules based on 1,4-Bis (9-phenyl-9*H*-carbazol-3-yl) bearing different phenyl cores. Through modifying different substitutes in phenyl cores, such as cyan as an electron-withdrawing group, and methyl and methoxy as electron donors, four small molecules were synthesized. Their photophysical properties were investigated in detail with the aim of understanding structure–property relationships and providing distinct information for further developing novel carbazole-based ultraviolet molecules. 

## 2. Experimental Section

### 2.1. Characterization and Instrumentation

All of the reagents were utilized as received from commercial sources. The solvents used in this study were further purified via standard methods.

^1^H- and ^13^C-nuclear magnetic resonance (NMR) spectra were performed on a Brüker AVANCE 400M spectrometer (Bruker, Billerica, MA, USA) by using deuterated chloroform solution. Time-of-flight (TOF) mass spectra were tested on a Bruker BIFLEXIII (Bruker, Billerica, MA, USA). Elemental analyses were carried out on an Elementar Vario (Elementar, Langenselbold, Germany). Differential scanning calorimetry (DSC) was carried out on a Netzsch DSC-204 (Netzsch, Selb, Germany) under nitrogen atmosphere with heating and cooling rates of 10 °C·min^−1^ and 70 °C·min^−1^, respectively. The thermogravimetric analyses (TGA) were tested on a TA TGA Q50 instrument (TA Instruments, New Castle, DE, USA) with a heating rate of 10 °C·min^−1^. The results of the UV-vis spectra were obtained from a PerkinElemer LAMBDA spectrophotometer (PerkinElemer, Waltham, MA, USA). The photoluminescence (PL) spectra were performed via using a PerkinElmer LS-45 fluorescence spectrophotometer (PerkinElemer, Waltham, MA, USA). The fluorescent quantum yields were recorded in dichloromethane at 297 K with quinine (0.05 mol·L^−1^ sulfate) as reference. Cyclic voltammetry (CV) measurements were performed at Princeton Applied Research PARSTAT 2273 (AMETEK Inc., Berwyn, PAa, USA) with a scan rate of 50 mV·s^−1^ under nitrogen, in which the tetra(n-butyl)ammoniumhexafluorophosphate (n-Bu_4_NPF_6_, 0.1 M) in acetonitrile or dichloromethane was used as the supporting electrolyte, and the conventional three-electrode configuration was utilized, consisting of glassy carbon as the work electrode, saturated calomel as reference electrode and platinum plate as counter electrode, respectively.

### 2.2. Synthesis of Targeting Molecules

#### 2.2.1. Synthesis of 1,4-Bis(9-phenyl-9*H*-carbazol-3-yl)benzene (CzP-H)

A mixture of *N*-phenyl-3-carbazolylboronic acid (1.25 g, 4.4 mmol) and 1,4-Dibromo benzene (0.475 g, 2 mmol) was added to a three-necked flask and dissolved in freshly distilled toluene (50 mL) under a nitrogen atmosphere. K_2_CO_3_ aqueous solution (2 M, 5 mL) was then added. Pd(PPh_3_)_4_ (0.12 g) was added in one portion to the reaction mixture, which was then heated (99 °C) and refluxed for 24 h. The mixture was cooled to room temperature, concentrated, and extracted with chloroform. The organic layer was purified using column chromatography on a silica gel with petroleum ether/dichloromethane (2:1) as the eluent to give CzP-H (0.896 g, 80% yield) as a white powder (melting point (m.p.) 239°C). ^1^H-NMR (CDCl_3_, 400 MHz, δ): 7.30~7.33 (1H, m); 7.43~7.44 (2H, t); 7.47~7.51 (2H, d); 7.60~7.66 (4H, d); 7.72~7.75 (1 H, d); 7.84 (2H, s); 8.21~8.23 (1H, s); 8.43 (1H, s). ^13^C-NMR (CDCl_3_, 400 MHz, δ): 76.68, 76.99, 77.31; 109.94; 110.07; 118.66; 120.09; 120.39; 123.55; 123.99; 125.40; 126.14; 127.12; 127.53; 127.67; 129.92; 133.16; 137.76; 140.25; 140.46; 141.44. MALDI-TOF: *m*/*z* 560.3 (*M^+^*). Anal. Calcd for C_42_H_28_N_2_: C 89.97, H 5.03, N 5.00; found: C 89.58; H 5.12; N 4.69.

#### 2.2.2. Synthesis of 2,5-bis(9-phenyl-9H-carbazol-3-yl)terephthalonitrile (CzP-CN)

A mixture of *N*-phenyl-3-carbazolylboronic acid (1.25 g, 4.4 mmol) and 2,5-dibromoterephthalonitrile (0.567 g, 2 mmol) was added to a three-necked flask with toluene (50 mL) under the protection of nitrogen atmosphere. The Pd(PPh_3_)_4_ (0.12 g) and K_2_CO_3_ aqueous solutions (2 M, 5 mL) were added in one portion, respective. Then, the reaction system was heated (99 °C) and refluxed for 24 h. The mixture was then cooled to room temperature, and extracted with dichloromethane three times. The crude products were purified by column chromatography with petroleum ether/dichloromethane (2:1) as the eluent to give CzP-CN (0.854 g, 70% yield) as a green solid (m.p. 351 °C). ^1^H-NMR (CDCl_3_, 400 MHz, δ): 7.34~7.37 (2H, t); 7.43~7.50 (4H, t); 7.52~7.57 (4H, t); 7.60~7.69 (10H, m); 8.08 (2H, s); 8.22 (1H, s); 8.24 (1H, s); 8.39 (2H, s). MALDI-TOF: *m*/*z* 610.3 (*M^+^*). Anal. Calcd for C_4__4_H_2__6_N_4_: C 86.53, H 4.29, N 9.17; found: C 85.28; H 4.23; N 8.89.

#### 2.2.3. Synthesis of 3,3′-(2,5-dimethyl-1,4-phenylene)bis(9-phenyl-9*H*-carbazole) (CzP-Me)

A mixture of *N*-phenyl-3-carbazolylboronic acid (1.25 g, 4.4 mmol) and 1,4-dibromo-2,5-dimethylbenzene (0.524 g, 2 mmol) was added to a three-necked flask with toluene (50 mL) under the protection of nitrogen atmosphere. The Pd(PPh_3_)_4_ (0.12 g) and K_2_CO_3_ aqueous solutions (2 M, 5 mL) were added in one portion, respectively. Then, the reaction system was heated (99 °C) and refluxed for 24 h. The mixture was cooled to room temperature, and extracted with dichloromethane three times. The crude products were purified using column chromatography on a silica gel with petroleum ether/dichloromethane (2:1) as the eluent to give CzP-Me (0.965 g, 82% yield) as a white powder (m.p. 277 °C). ^1^H-NMR (CDCl_3_, 400 MHz, δ): 2.39 (3H, s); 7.30~7.34 (2H, d); 7.43~7.50 (5H, m); 7.62 (2H, s); 7.64 (2H, s); 8.16 (1H, s); 8.17 (1H, s). ^13^C-NMR (CDCl_3_, 400 MHz, δ): 20.18; 76.67; 76.99; 77.31; 109.27; 109.87; 119.99; 120.31; 120.82; 123.47; 123.31; 126.02; 127.12; 127.47; 129.89; 132.34; 132.93; 133.81; 137.83; 139.93; 141.23; 141.31. MALDI-TOF: *m*/*z* 588.4 (*M^+^*). Anal. Calcd for C_4__4_H_32_N_2_: C 89.76, H 5.48, N 4.76; Found: C 88.21; H 5.30; N 4.49.

#### 2.2.4. Synthesis of 3,3′-(2,5-dimethoxy-1,4-phenylene)bis(9-phenyl-9*H*-carbazole) (CzP-OMe)

A mixture of *N*-phenyl-3-carbazolylboronic acid (1.25 g, 4.4 mmol) and 1,4-dibromo-2,5-dimethoxybenzene (0.588 g, 2 mmol) was added to a three-necked flask with toluene (50 mL) under the protection of nitrogen atmosphere. The Pd(PPh_3_)_4_ (0.12 g) and K_2_CO_3_ aqueous solutions (2 M, 5 mL) were added in one portion, respectively. Then, the reaction system was heated (99 °C) and refluxed for 24 h. The mixture was cooled to room temperature, and extracted with dichloromethane three times. The crude products were purified using column chromatography on a silica gel with petroleum ether/dichloromethane (2:1) as the eluent to give CzP-OMe (0.992 g, 80% yield) as a white solid (m.p. 297 °C). ^1^H-NMR (CDCl_3_, 400 MHz, δ): 3.85 (3H, t); 7.16 (1H, s); 7.25 (1H, s); 7.42~7.44 (2H, d); 7.46~7.49 (2H, d); 7.61~7.65 (4H, d); 7.78~7.70 (1H, d); 8.20 (1H, s); 8.37 (1H, s). ^13^C-NMR (CDCl_3_, 400 MHz, δ): 56.71; 76.68; 77.00; 77.31; 109.43; 109.83; 115.46; 119.96; 120.40; 121.14; 123.40; 123.59; 125.94; 127.12; 127.46; 127.77; 129.88; 130.36; 130.84; 137.80; 140.16; 141.29. MALDI-TOF: *m*/*z* 620.4 (*M^+^*). Anal. Calcd for C_44_H_32_N_2_O_2_: C 85.14, H 5.20, N 4.51; Found: C 85.17; H 5.25; N 4.24.

## 3. Results and Discussion

### 3.1. Synthesis

**Scheme 1:**
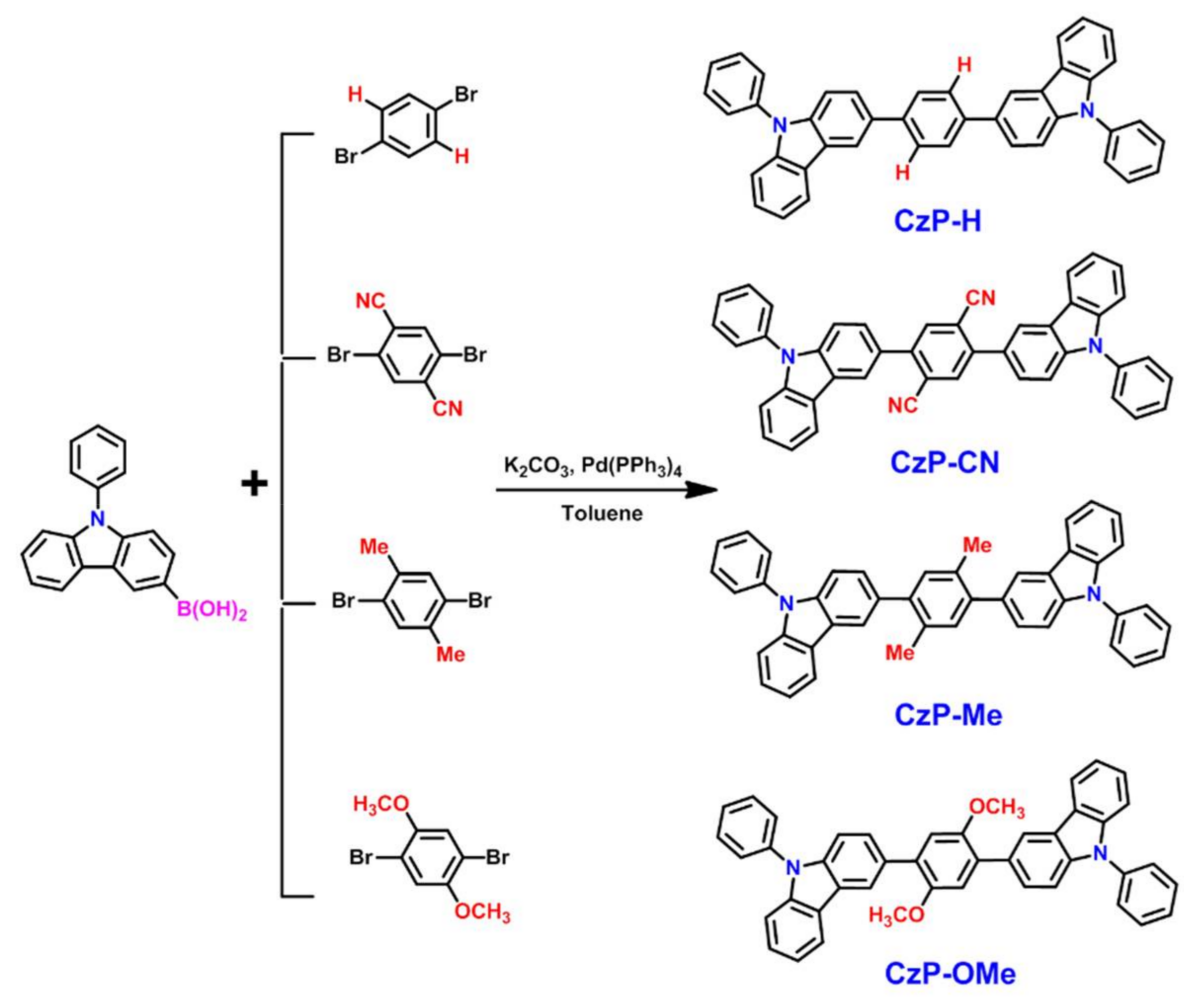
Synthetic routes of the CzP-H, CzP-CN, CzP-Me, and CzP-OMe.

Scheme 1 shows the detailed synthetic procedures for CzP-H, CzP-CN, CzP-Me, and CzP-OMe. All of the desired compounds were readily obtained by one-pot Suzuki coupling reaction via using the commercially available *N*-phenyl-3-carbazolylboronic acid and 1,4-dibromo benzene derivatives directly. To promote the reaction yields and simplify the procedures of obtaining target compounds, the previous reaction systems of tetrabutylammonium bromide (TBAB), PdCl_2_, and the solvent of acetone were replaced by Pd(pph_3_)_4_ and toluene, respectively [29,30]. These improvements could successfully control the reaction to get the target products with high yields (>70%). The CzP-H, CzP-Me and CzP-OMe are readily dissolved in common organic solvents such as toluene, chloroform, THF and chlorobenzene. However, the CzP-CN is poorly soluble in these solvents, which is possibly resulting from the introduction of electron-withdrawing cyano groups that the intermolecular force are stronger in CzP-CN than in CzP-H, CzP-Me and CzP-OMe.

### 3.2. Thermal Stability

**Figure 1:**
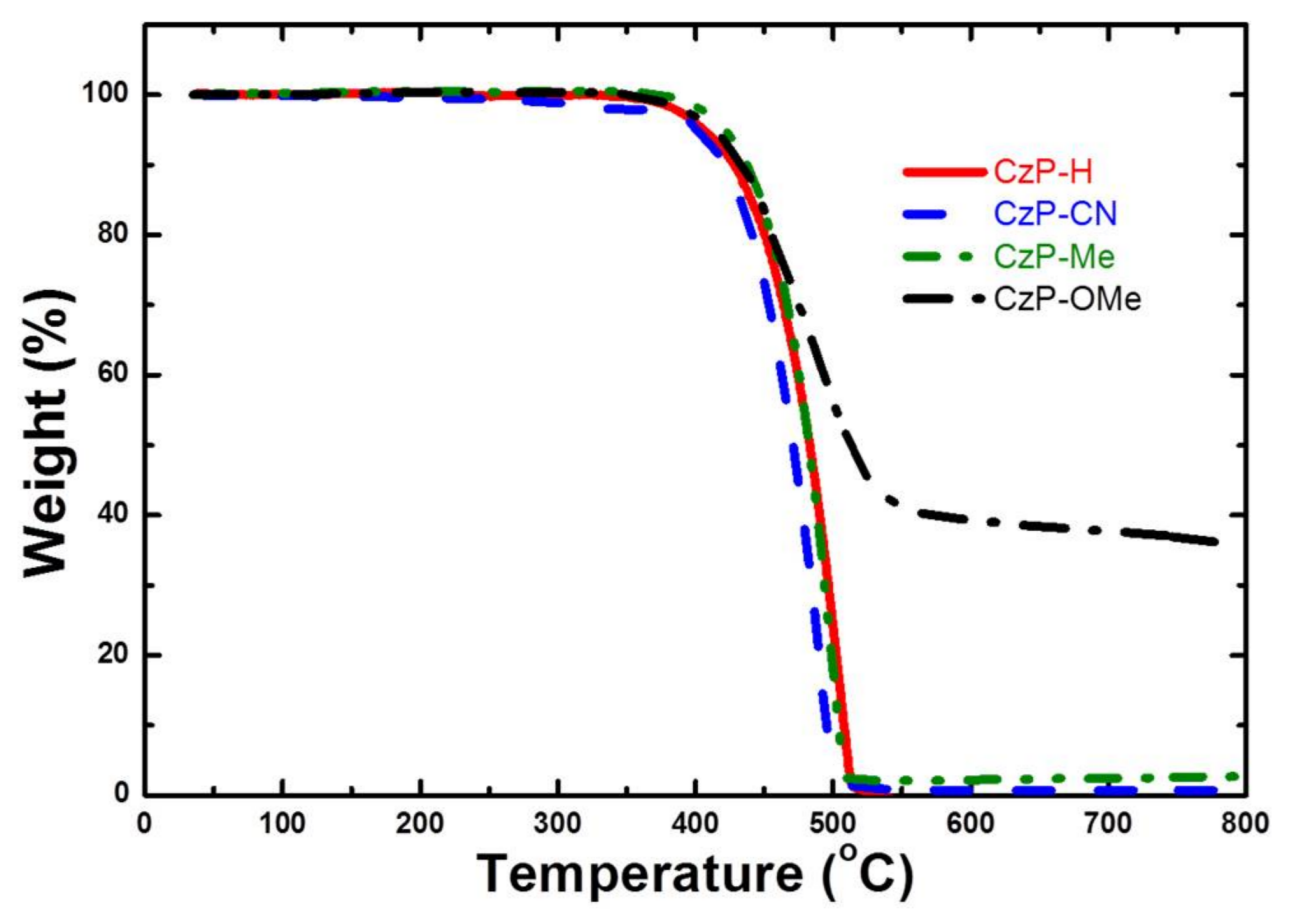
Thermogravimetric analysis (TGA) curves for CzP-H, CzP-CN, CzP-Me, and CzP-OMe.

The thermal properties of CzP-H, CzP-CN, CzP-Me, and CzP-OMe were evaluated by thermogravimetric analysis (TGA), respectively. The pertinent data are summarized in Table 1. In Figure 1a, the TGA test suggests that all of these small molecules exhibit excellent thermal stabilities, with decomposition temperatures (*T*_d_) at 5% weight loss ranging from 403 °C to 421 °C. On the basis of the thermal properties, all of these four small molecules show very high thermal stability, which is significantly beneficial for realizing stable ultraviolet materials.

### 3.3. Theoretical Calculation

**Figure 2:**
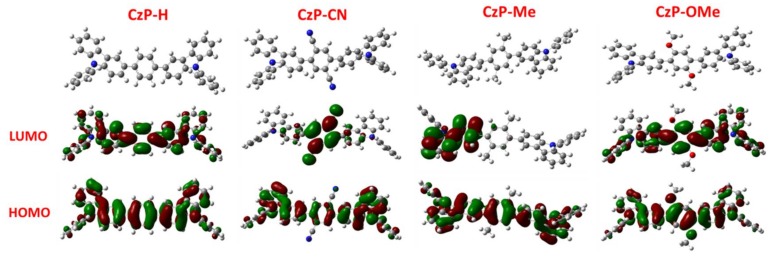
Highest occupied molecular orbitals (HOMOs) and lowest unoccupied molecular orbitals (LUMOs) for CzP-H, CzP-CN, CzP-Me, and CzP-OMe by density functional theory (DFT) calculations at the B3LYP/6-31G(d) level.

To understand the electronic properties theoretically, the density functional theory (DFT) calculations were carried out to analyze these four small molecules via using the Gaussian 09 program at the B3LYP/6-31G(d) level, and the relative electronic structures are presented in Figure 2. For CzP-H, CzP-CN, CzP-Me, and CzP-OMe, the highest occupied molecular orbitals (HOMOs) and lowest unoccupied molecular orbital (LUMOs) were spread out the entire molecule backbones with distinct locations, indicating that the different substitutes in the phenyl cores could influence the distribution of electron density in the carbazole-based backbones. The HOMO orbitals of these four compounds reside much more intensively on both carbazole and phenyl cores, which is possibly due to the π-bridge effect on symmetric carbazole units. However, the LUMO orbitals are quite different from the HOMOs. Compared with CzP-H, it can be clearly observed that the LUMOs reside on the phenyl core, with a cyano group for CzP-CN, resulting from the strong electron-withdrawing properties of the cyano groups. For CzP-Me and CzP-OMe, the substitutes in the phenyl core have similar electron-donating groups, but the LUMO state of CzP-Me is only localized on one carbazole unit, while the LUMO state of CzP-OMe is the closest to CzP-H. This is possibly because the methoxyl group in CzP-OMe is much more flexible than the methyl in CzP-Me, resulting in the more planar structure of CzP-OMe than CzP-Me. The distinction of the electron density of the HOMOs and LUMOs implies that the different substitutes in the phenyl cores can successfully change the twist angle of molecular structure, even though the π-bridge effect or intermolecular interactions could be influenced. Hence, the molecular orbital analysis clearly indicates that the substituents at the 2,4-positions of the phenyl cores alter the energy levels of the symmetric carbazole-based small molecules.

### 3.4. Photophysical Properties

**Figure 3:**
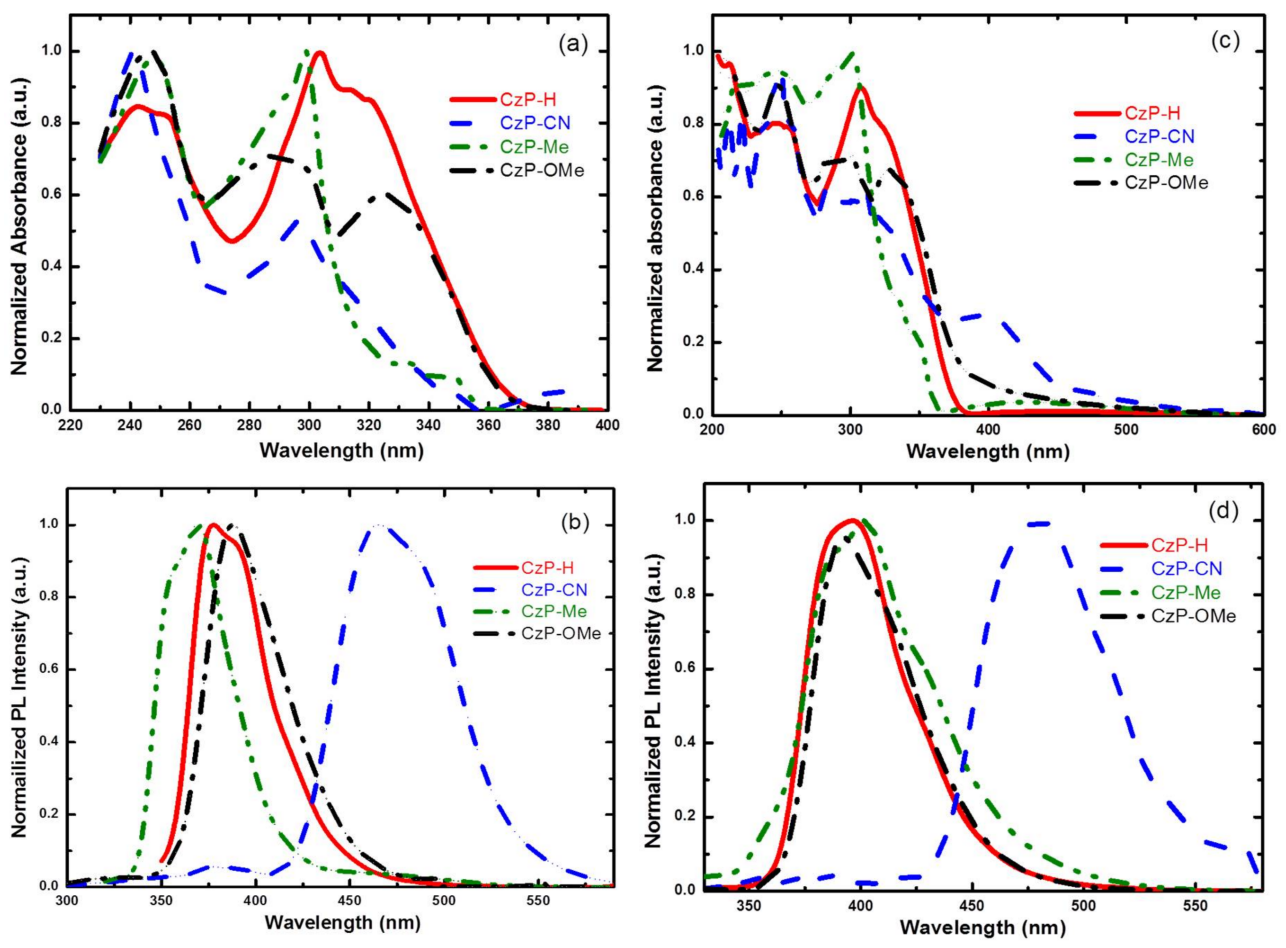
(**a**) Ultraviolet-visible and (**b**) PL spectra of CzP-H, CzP-CN, CzP-Me, and CzP-OMe in DCM solvent; (**c**) Ultraviolet-visible and (**d**) PL spectra of CzP-H, CzP-CN, CzP-Me, and CzP-OMe in films.

The UV-visible absorption and PL spectra of CzP-H, CzP-CN, CzP-Me, and CzP-OMe in solution and film are shown in Figure 3, and the relative data are summarized in Table 2. For the absorption spectra of CzP-H, CzP-CN, CzP-Me and CzP-OMe in dichloromethane (DCM), the maximal absorption can be detected at 243 nm and 304 nm for CzP-H, 241 nm and 296 nm for CzP-CN, 248 nm and 299 nm for CzP-Me, and 246 nm, 289 nm, and 325 nm for CzP-OMe, respectively. The absorption spectra in film are slightly different, with the maximal absorption at 212 nm and 308 nm for CzP-H, 220 nm, 250 nm and 412 nm for CzP-CN, 216 nm and 302 nm for CzP-Me, and 246, and 328 nm and 332 nm for CzP-OMe, respectively. In addition, the red-shifted absorption at 330 nm for CzP-OMe can be illustrated as the more planar structure than CzP-Me. From the intersection of normalized absorption and PL spectra in film, the *E*_00_ of CzP-H, CzP-CN, CzP-Me, and CzP-OMe were 3.39 eV, 2.83 eV, 3.50 eV, and 3.35 eV, respectively (Figure S12). These results show that CzP-Me has a larger band gap (*E_g_*) than CzP-H, where the CzP-CN has the smallest *E_g_*. The PL spectra in solution and in film show identical UV or deep-blue emissions, where the emission peaks are at 377 nm and 396 nm for CzP-H, 465 nm and 480 nm for CzP-CN, and 369 nm and 402 nm for CzP-Me, 388 nm and 392 nm for CzP-OMe in solution and in film, respectively. CzP-Me shows a shorter emission wavelength, which proves that the steric electron-donating methyl groups can successfully increase the twist angle in their molecule structure and reach the UV emission. The emission at 465 nm for CzP-CN in solution is probably caused by the intramolecular donor–acceptor charge-transfer state. The spectra in film are slightly red-shifted compared with those in solution; however, the bathochromic shift of CzP-Me is 33 nm and CzP-OMe is 4 nm, respectively. This possibly because the largest twist angle of CzP-Me in film has been restricted, and imposes less influence in the planar structure of CzP-OMe.

Compared with CzP-H, CzP-CN, and CzP-Me, the UV-vis absorption and PL spectra of CzP-CN are obviously different, and both of them show the greater red-shifted spectra, which is possibly because cyano is a strong electron-withdrawing unit that would form the obvious intramolecular charge-transfer state from the carbazole unit to CN-substituted phenyl moiety [36]. These phenomena are also illustrated by the theoretical computation. It can be seen that these four compounds have almost the same distributions of electron clouds in the HOMO energy levels. However, there are completely different distributions in the LUMOs. Furthermore, strong electron-donating carbazole units and strong electron-drawing cyano units turn CzP-CN into a donor–acceptor (D-A)-type blue emission, which would be a promising candidate of the blue fluorescent compound. The PL spectra of CzP-CN show a broad full width at half maximum (FWHM), which indicate that the D-A system extends π-conjugation, inducing a large bathochromic shift and impairing the color purity. In general, CzP-H, CzP-Me and CzP-OMe exhibit UV to blue-violet emission with maxima emission from 369 nm to 402 nm. As shown in Table 2, the photoluminescence quantum yield (PLQY) of CzP-H, CzP-Me, and CzP-OMe are 71%, 49%, and 35%, respectively. CzP-H shows the highest PLQY, and CzP-OMe shows the lowest one. Since CzP-CN is hard to solve in organic solvents, it is difficult to test the PLQY. Therefore, these as-prepared carbazole derivatives can be used potentially as excellent candidates for the blue-violet to UV OLEDs. Through considering these optical properties penetratingly, it is proposed that the absorption and PL spectra are affected by the properties of peripheral substituents. As shown in Figure 3, the absorption and PL spectra of CzP-CN in solution and in film are obviously red-shifting compared with CzP-H, CzP-Me, and CzP-OMe. The almost 100-nm red-shift in PL can be ascribed to the strong electron-withdrawing nature of cyano presented in CzP-CN. However, CzP-Me shows the nearly same absorption and PL spectra as the original compound CzP-H, and only a small blue-shift in PL spectra due to the steric structure via the introduction of methyl. Additionally, the capabilities of peripheral substituents (-H, -Me and -OMe) present little effect on the photophysical properties. 

### 3.5. Electrochemical Properties

**Figure 4:**
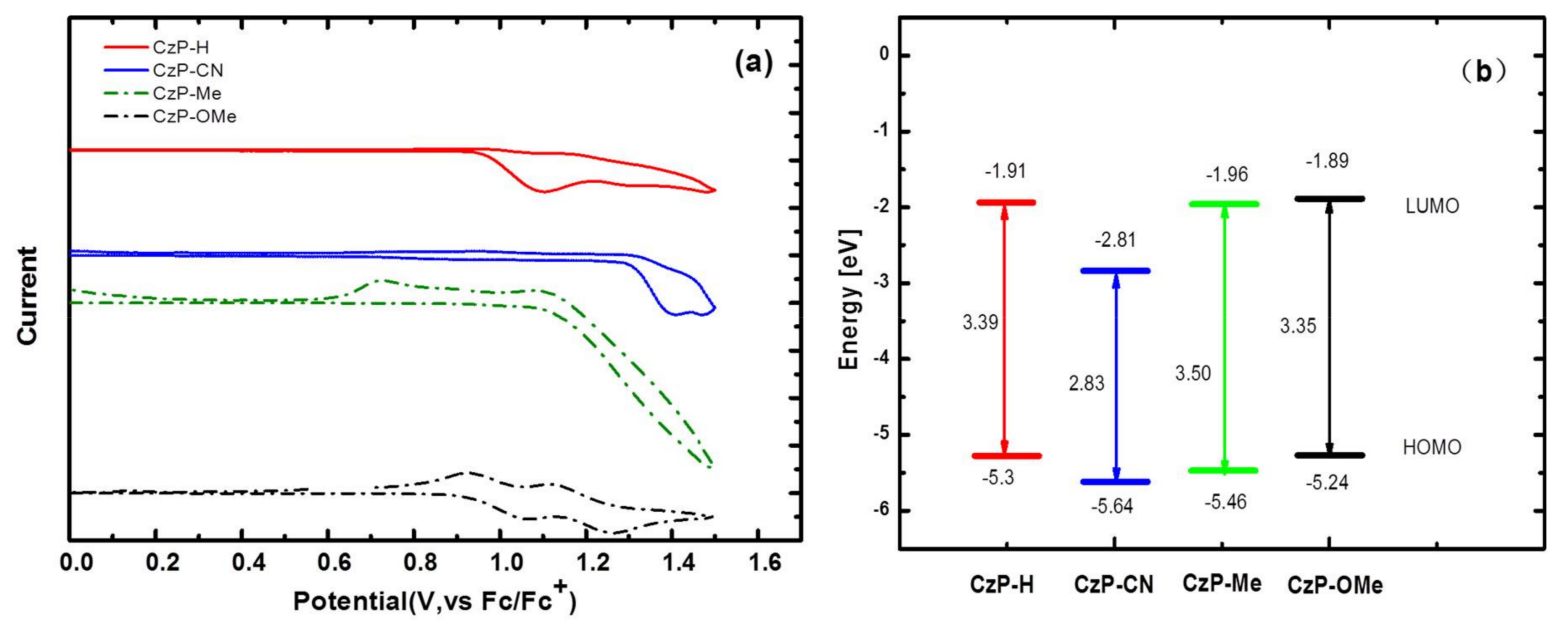
(**a**) Cyclic voltammogram curves of CzP-H, CzP-CN, CzP-Me, and CzP-OMe in solutions and (**b**) an energy-level diagram for CzP-H, CzP-CN, CzP-Me, and CzP-OMe.

The cyclic voltammetry measurements were used to study the electrochemical properties of these four small molecules. The ferrocene was used as a standard internal reference in this study, and the ferrocene/ferrocenium (Fc/Fc^+^) redox shows a half-wave potential (*E*_1/2_) at 0.47 V. The CV curves are presented in Figure 4, and the relative data are concluded in Table 3. The highest occupied molecular orbital (HOMO) energy levels were calculated according to the empirical formula *E*_HOMO_ = −e(*E*_ox_ + 4.8 − *E*_1/2,(Fc/Fc_^+^_)_) [30], where *E*_ox_ is the onset of the oxidation potential. From Figure 4, the *E*_ox_s are 0.97 eV, 1.31 eV, 1.13 eV and 0.91 eV for CzP-H, CzP-CN, CzP-Me and CzP-OMe, respectively. Through the calculation, the HOMOs of these four small molecules were −5.30 eV, −5.64 eV, −5.46 eV, and −5.24 eV for CzP-H, CzP-CN, CzP-Me and CzP-OMe, respectively. Since we can’t get the reductive curves from the CV test, the lowest unoccupied molecular orbital (LUMO) energy levels are calculated from the empirical equation of *E*_LUMO_ = *E*_HOMO_ + *E*_00_, where the *E*_00_ is the intersection of the normalized absorption spectra with the PL spectra. Through the calculation, the LUMOs of CzP-H, CzP-CN, CzP-Me, and CzP-OMe are −1.91 eV, −2.81 eV, −1.96 eV, and −1.89 eV, respectively. It shows that CzP-CN has the lowest HOMO level, indicating that the existence of the electron-withdrawing substituent (cyano) can decrease the HOMO energy levels. Alternatively, the HOMO of CzP-Me are 0.16 eV lower than the HOMO of CzP-H, but CzP-OMe is 0.06 eV higher than the HOMO of CzP-H. This is possibly because the methyl group will twist the molecular structure of CzP-Me, leading to the lower HOMO energy level, while the electron-donating methoxyl group could promote the HOMO level distinctly. These are in agreement with not only the results from the UV and PL spectra, but also the theoretical calculations. In addition, from the calculation data of LUMOs, these results can also well illustrate that CzP-CN has the lowest LUMO energy level, and CzP-OMe has the highest. It implies that the strong CN groups can form the charge-transfer state efficiently; however, the methyl groups may twist and restrict the conjugation of carbazoles to generate UV light emission. 

## 4. Conclusions

Through chemical substitution, four small molecules based on carbazole derivatives with symmetrical configurations anchoring different peripheral substituents, such as methyl, methoxyl, and nitrile, were investigated in detailed. These four compounds have high melting points and thermal decomposition temperatures, which demonstrates that they possess very high thermal stabilities. Theoretical calculations, photophysical properties, and electrochemical properties suggest that electron-donating groups will increase the HOMO energy levels, and electron-withdrawing units could lower the LUMO energy levels efficiently in these kinds of molecular configurations with different peripheral substituents. In addition, the PL spectra of these compounds display UV to blue-violet fluorescence (λ_max_= 392–402 nm) in films with narrow FWHM (30–45 nm). Furthermore, the CzP-H shows the highest PLQY of 72%, and CzP-Me gives the shortest fluorescent emission of 392 nm with a PLQY of 50%. Therefore, the introduction of π bridges with different substitutes between two carbazole units would affect the photophysical properties efficiently. These results indicate that the prepared carbazole derivatives would be potentially versatile candidates for constructing high-efficiency UV and blue-violet organic light emitting materials.

## Figures and Tables

**Table 1:** Thermal properties of CzP-H, CzP-CN, CzP-Me and CzP-OMe.

Compound	*T*_m_ (°C)	*T*_d_ (°C)
CzP-H	239	406
CzP-CN	351	403
CzP-Me	277	421
CzP-OMe	297	413

**Table 2:** Photophysical properties of the compounds.

Compound	λ_abs,DCM_ (nm)	λ_abs,film_ (nm)	λ_PL,DCM_ (nm)	λ_PL,film_ (nm)	*E*_00_ (eV)	*Φ*_f_ (%)
CzP-H	243, 304	212, 308	377	396	3.39	71
CzP-CN	241, 296	220, 250, 412	465	480	2.83	-
CzP-Me	248, 299	216, 302	369	402	3.50	49
CzP-OMe	246, 289, 325	246, 328, 332	388	392	3.35	35

**Table 3:** Electrochemical properties of the compounds.

Compound	*E*_ox_ (V)	*E*_HOMO_ (eV)	*E*_LUMO_ (eV)
CzP-H	0.97	−5.30	−1.91
CzP-CN	1.31	−5.64	−2.81
CzP-Me	1.13	−5.46	−1.96
CzP-OMe	0.91	−5.24	−1.89

## Supplementary Materials

The following are available online at http://www.mdpi.com/1996-1944/11/4/617/s1. Figure S1: ^1^H NMR spectrum of CzP-H; Figure S2: ^1^H NMR spectrum of CzP-CN; Figure S3: ^1^H NMR spectrum of CzP-Me; Figure S4: ^1^H NMR spectrum of CzP-OMe; Figure S5: ^13^C NMR spectrum of CzP-H; Figure S6: ^13^C NMR spectrum of CzP-Me; Figure S7: ^13^C NMR spectrum of CzP-OMe; Figure S8: HR-MS spectrum of CzP-H; Figure S9: HR-MS spectrum of CzP-CN; Figure S10: HR-MS spectrum of CzP-Me; Figure S11: HR-MS spectrum of CzP-OMe; Figure S12: Normalized absorption spectra and normalized PL spectra.
