# Supplementary material for: π-Bridge Effect on Symmetric Carbazole-Based Small Molecules for Realizing Ultraviolet Fluorescent Emission
Source: Materials (Basel). 2018 Apr 17;11(4):617. doi: 10.3390/ma11040617 (PMC5951501; doi:10.3390/ma11040617)
Supplement: Supplementary file 1 [file materials-11-00617-s001.pdf]

Supplementary Material

π-Bridge Effect on Symmetric Carbazole-Based Small Molecules for Realizing Ultraviolet Emission

Siyang Liu, [1] Pengju Lin, [1] Fangfang Niu, [1] Pengju Zeng, [1,*] and Bin Zhang[1,2,*]

1 Key Laboratory of Optoelectronic Devices and Systems of Ministry of Education and Guangdong Province, College of Optoelectronic Engineering, Shenzhen University, Shenzhen 518060, P. R. China

2 School of Materials Science and Engineering, Jiangsu Engineering Laboratory of Light-Electricity-Heat Energy-Converting Materials and Applications, Jiangsu Key Laboratories of Environment-Friendly Polymers, National Experimental Demonstration Center for Materials Science and Engineering, Changzhou University, Changzhou 213164, P. R. China

* Correspondence: zengpj@szu.edu.cn (P.Z.); msbinzhang@outlook.com (B.Z.)

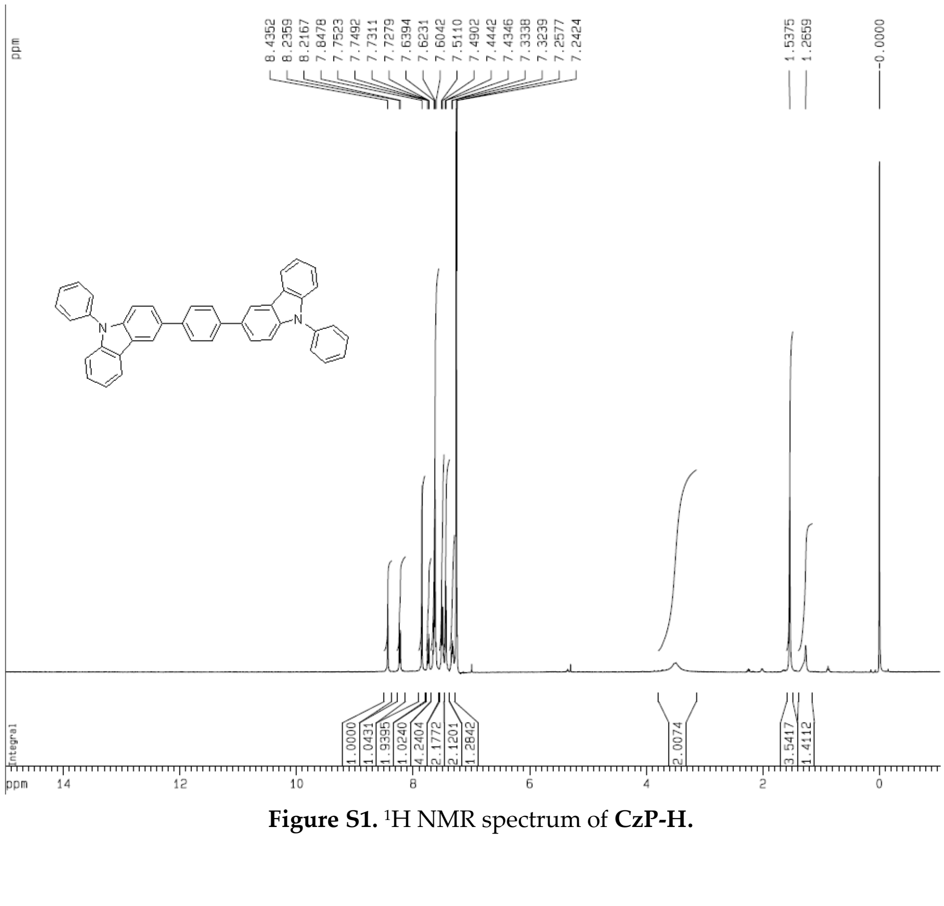

Figure S1. ^{1}H NMR spectrum of **CzP-H.**

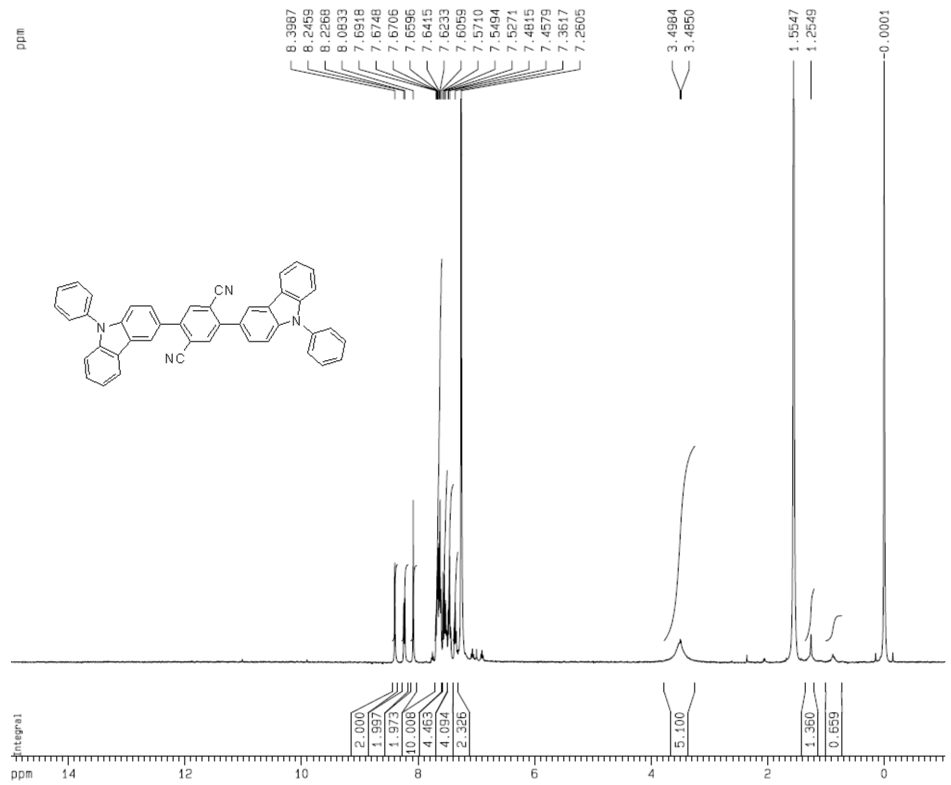

Figure S2. ^{1}H NMR spectrum of **CzP-CN**.

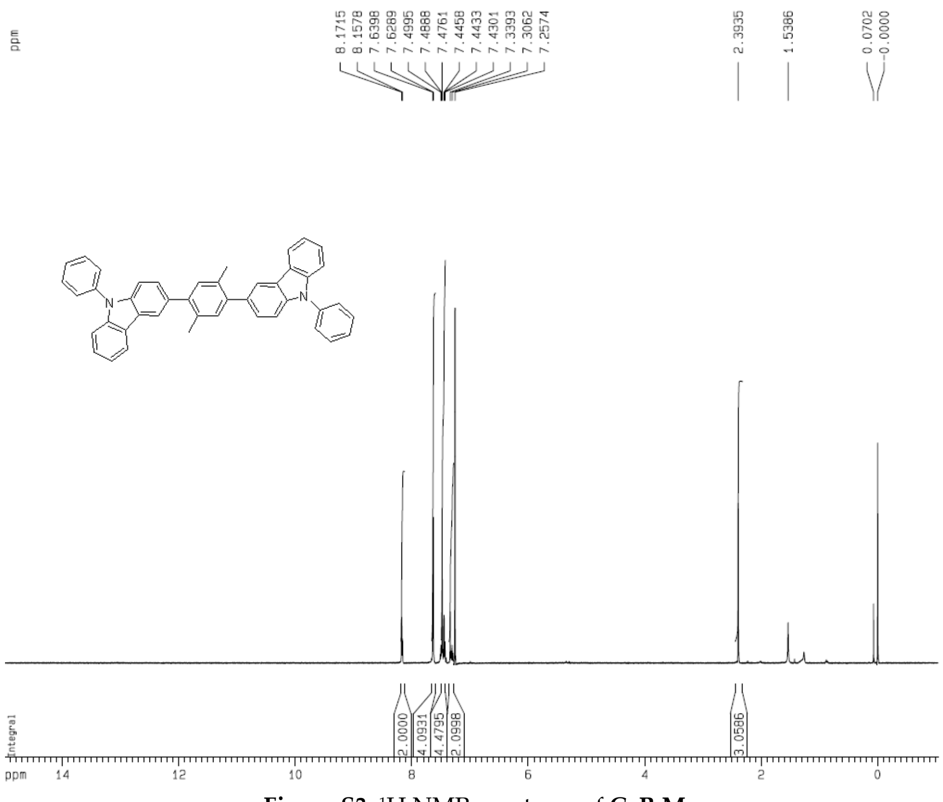

Figure S3. ^{1}H NMR spectrum of **CzP-Me**.

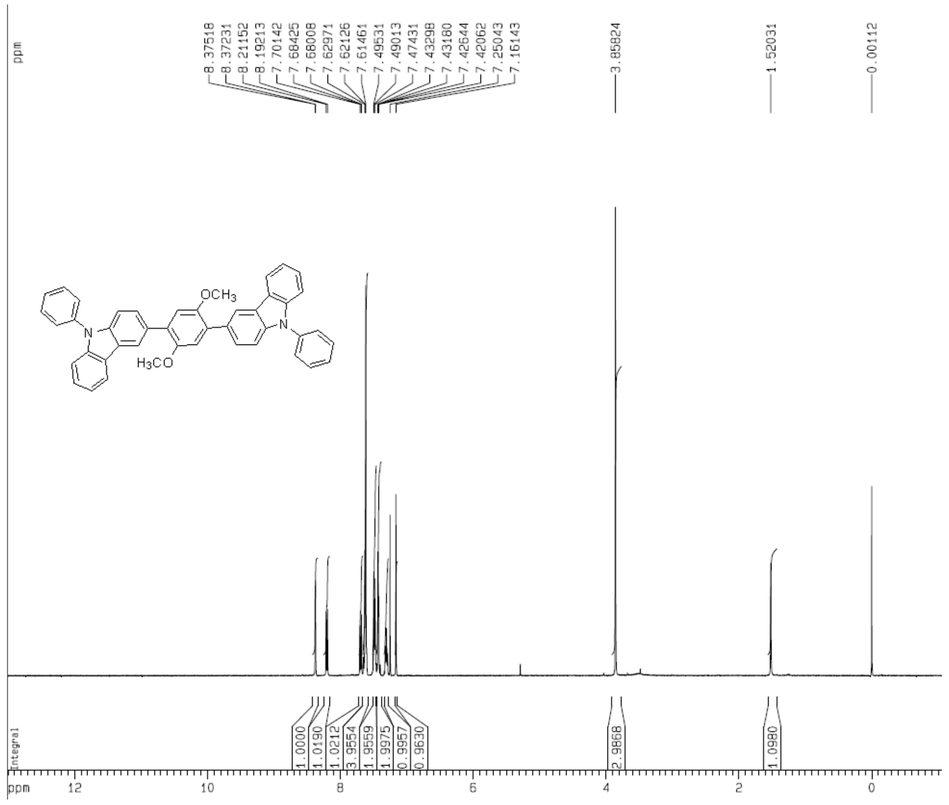

Figure S4. ^{1}H NMR spectrum of **CzP-OMe**.

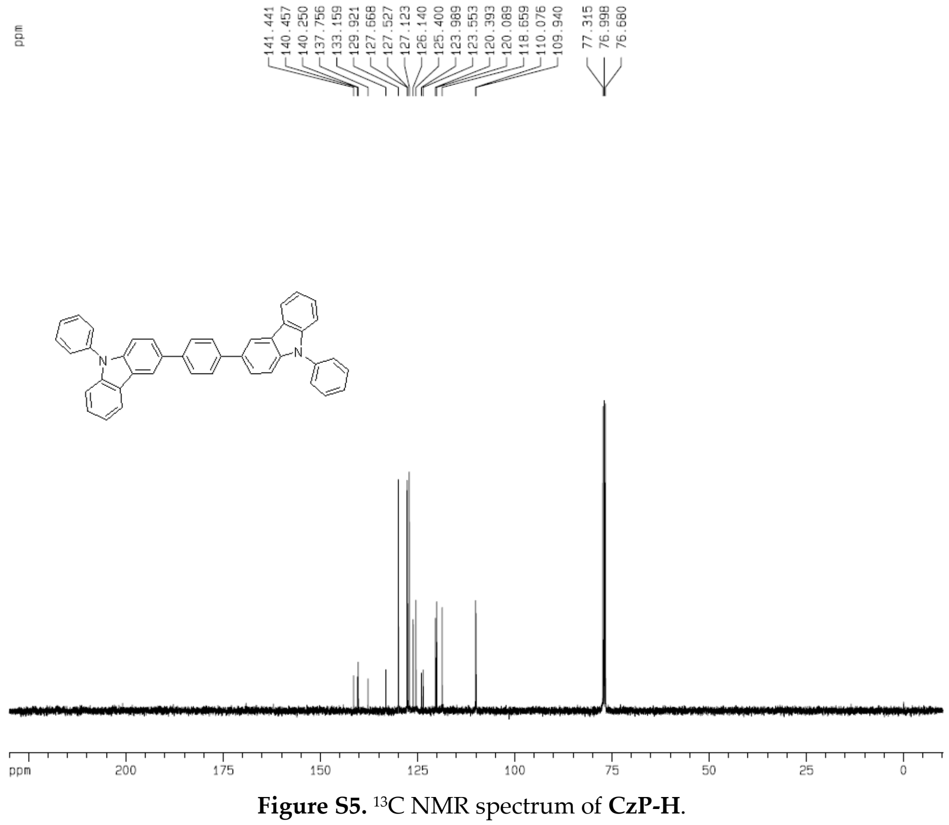

Figure S5. ^{13}C NMR spectrum of **CzP-H**.

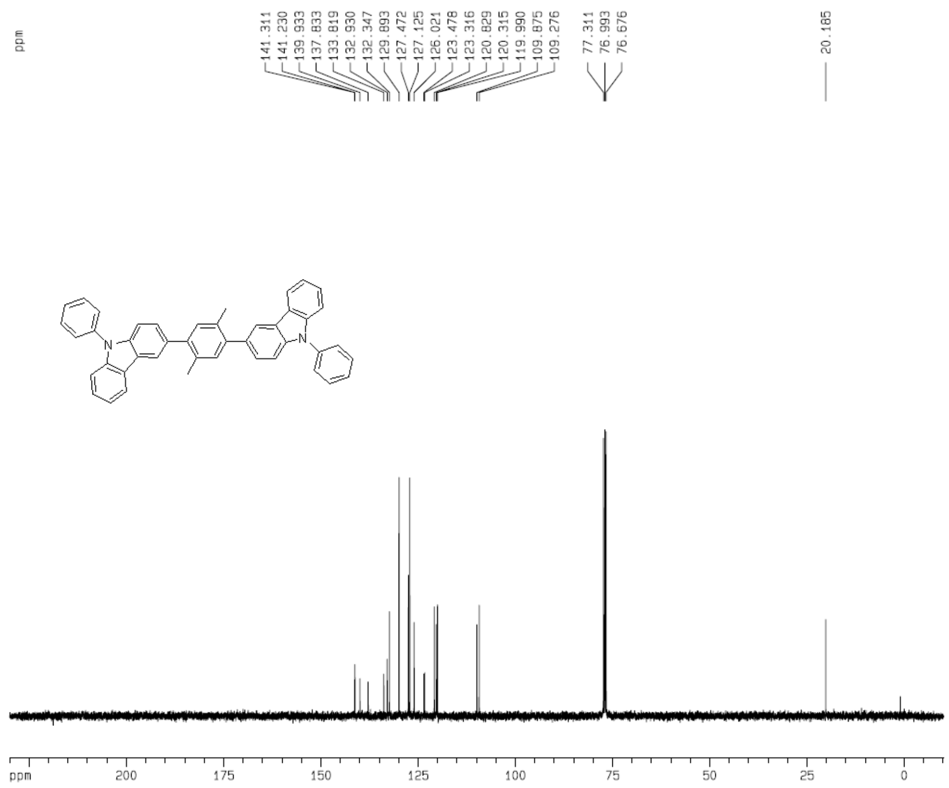

Figure S6. ^{13}C NMR spectrum of **CzP-Me**.

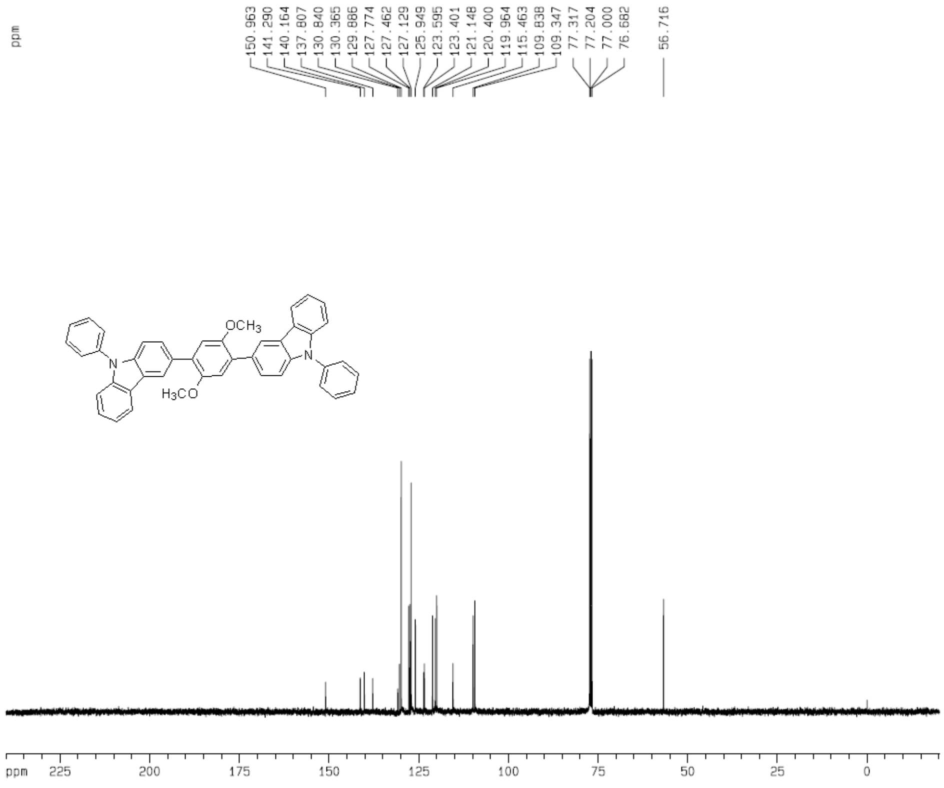

Figure S7. ^{13}C NMR spectrum of **CzP-OMe**.

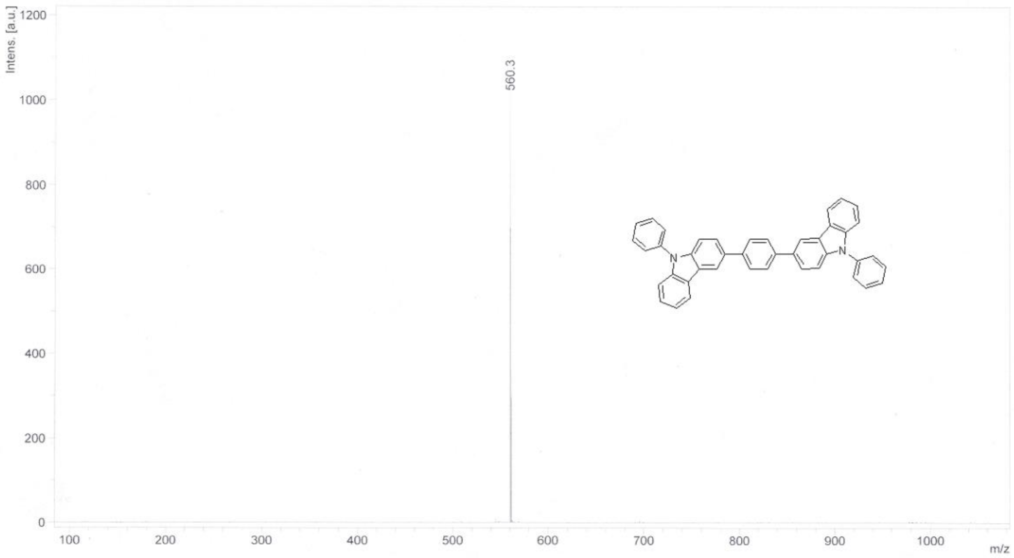

Figure S8. HR-MS spectrum of **CzP-H**.

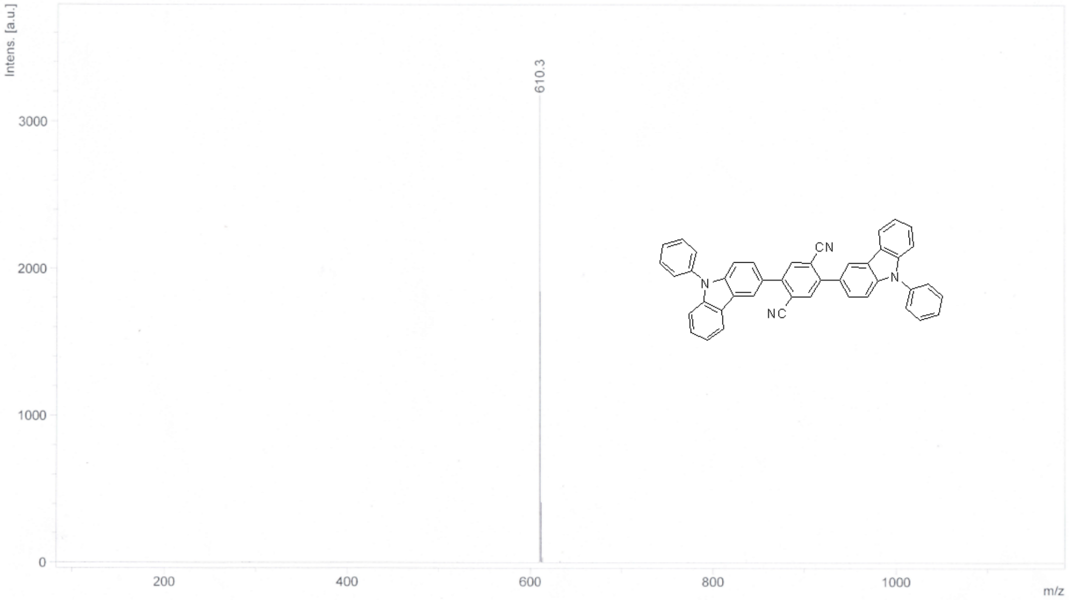

Figure S9. HR-MS spectrum of **CzP-CN**.

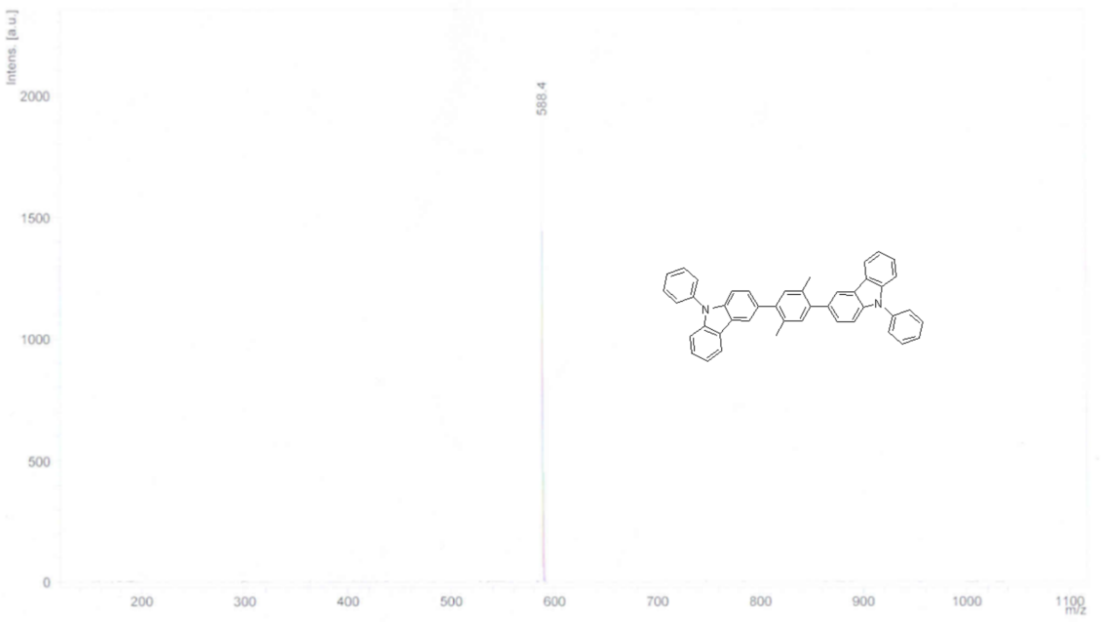

Figure S10. HR-MS spectrum of **CzP-Me**.

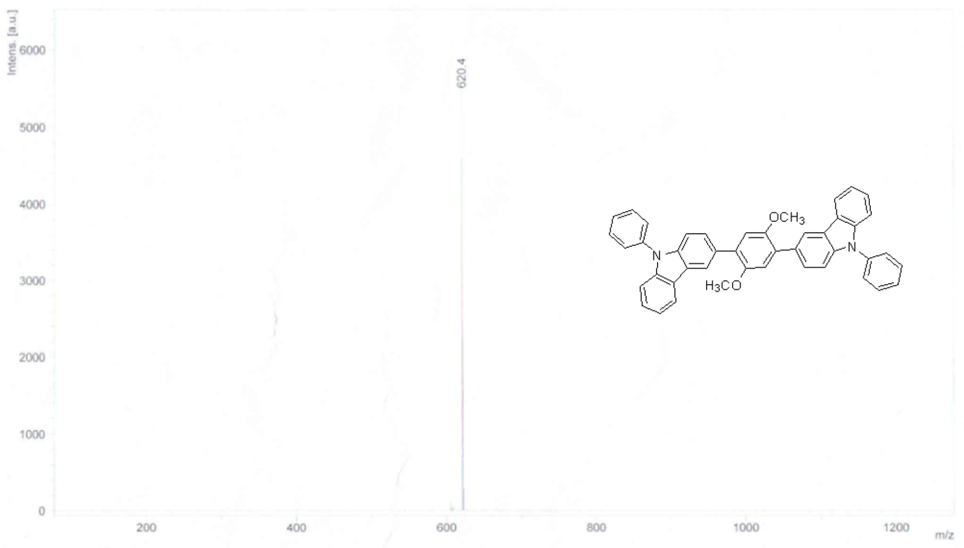

Figure S11. HR-MS spectrum of **CzP-OMe**.

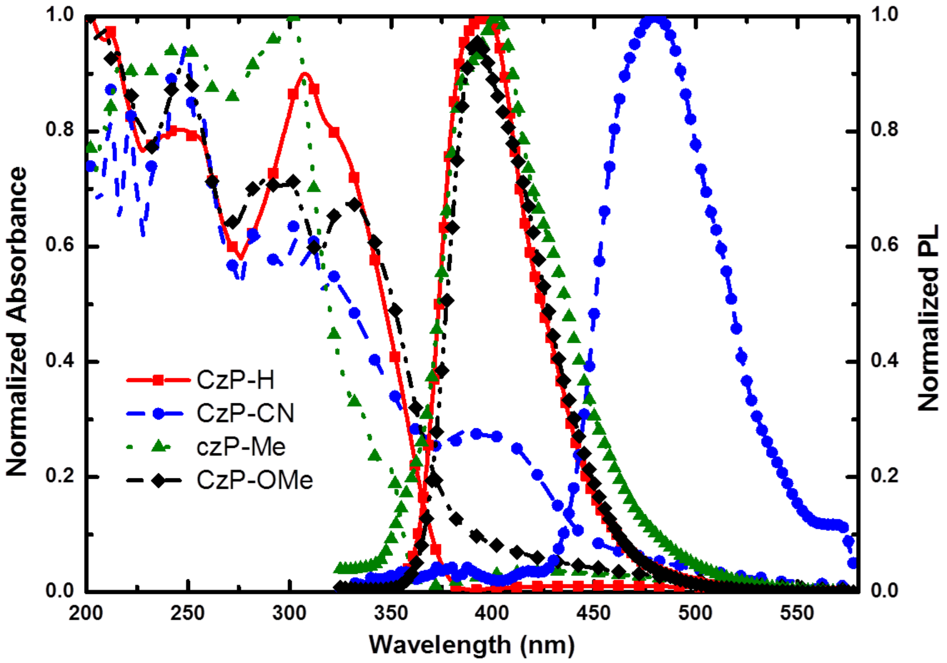

Figure S12. Normalized absorption spectra and normalized PL spectra.
